# Potentiometric Studies on Ion-Transport Selectivity in Charged Gold Nanotubes

**DOI:** 10.3390/nano14141209

**Published:** 2024-07-16

**Authors:** Thomas T. Volta, Stevie N. Walters, Charles R. Martin

**Affiliations:** Department of Chemistry, University of Florida, Gainesville, FL 32611-7200, USA

**Keywords:** nanotubes, nanotube membranes, nanoionics, nanofluidics, potentiometry, permselectivity, Debye theory

## Abstract

Under ideal conditions, nanotubes with a fixed negative tube-wall charge will reject anions and transport-only cations. Because many proposed nanofluidic devices are optimized in this ideally cation-permselective state, it is important to know the experimental conditions that produce ideal responses. A parameter called C_crit_, the highest salt concentration in a contacting solution that still produces ideal cation permselectivity, is of particular importance. Pioneering potentiometric studies on gold nanotubes were interpreted using an electrostatic model that states that C_crit_ should occur when the Debye length in the contacting salt solution becomes equivalent to the tube radius. Since this “double-layer overlap model” (DLOM), treats all same-charge ions as identical point charges, it predicts that all same-charged cations should produce the same C_crit_. However, the effect of cation on C_crit_ in gold nanotubes was never investigated. This knowledge gap has become important because recent studies with a polymeric cation-permselective nanopore membrane showed that DLOM failed for every cation studied. To resolve this issue, we conducted potentiometric studies on the effect of salt cation on C_crit_ for a 10 nm diameter gold nanotube membrane. C_crit_ for all cations studied were, within experimental error, the same and identical, with values predicted by DLOM. The reason DLOM prevailed for the gold nanotubes but failed for the polymeric nanopores stems from the chemical difference between the fixed negative charges of these two membranes.

## 1. Introduction

The emerging field of nanofluidics promises not only a deeper understanding of the properties of nanoscale-confined solutions [1,2], but also new nanopore/tube/channel devices for alternative energy [3,4,5,6], water desalinization [7,8,9], solution pumping [10,11], nanofluidic computing [12], and other applications [13,14,15,16]. Many of these proposed applications are optimized when the nanoconfined solution transports ions of only one sign, most often cations and not anions. This ideally cation-permselective/cation-conducting state can be obtained by attaching anionic chemical functionalities, e.g., carboxylates on carbon nanotubes [13,14], silicates on the walls of Si-based nanochannels [15,16,17], and adsorbed chloride on gold nanotubes [18,19]. Ideally, these anionic groups would prohibit anions and salt from entering the confined solution, yielding the ideal cation-permselective state.

Because the efficiencies of many of the proposed nanopore/tube/channel devices are optimized in this ideal cation-conducting state [4,6], it is imperative to know the experimental conditions that produce ideal responses [18,19,20,21]. Potentiometric concentration cells, where a nanopore/tube membrane separates salt solutions of differing concentrations, have been used to explore permselectivity in confined solutions [18,20,22]. These cells convert the transmembrane salinity differences to a membrane voltage, E_mem_, which is maximized for the ideal state and diminished if ideality cannot be achieved.

Of particular importance is a parameter called C_crit_, the highest salt concentration in the contacting solution that still produces ideal cation permselectivity (anion and salt rejection) in the tube-confined solution [20]. Pioneering studies on gold nanotube membranes suggested that C_crit_ was inversely proportional to the inside diameters of nanotubes [18,19,23]. These, admittedly scant [18], data were interpreted using an electrostatic model that states that C_crit_ should occur when an electrolyte-solution parameter called the Debye length [24,25] becomes equivalent to the tube radius. Since this model, sometimes called the double-layer overlap model (DLOM), treats all same-charge ions as identical point charges, it predicts that all same-charged cations should produce the same value of C_crit_. While DLOM is widely accepted, this hypothesis has never been experimentally demonstrated because all prior potentiometric studies of gold nanotube membranes used only potassium ions.

We have, however, recently investigated the effect of cation on C_crit_ for a polymeric nanopore membrane with 10 nm pores and a negative pore-wall charge due to carbonate groups [20]. In contradiction to DLOM, C_crit_ was found to vary dramatically with cation; for example, C_crit_ for Cs^+^ was 20-times higher than C_crit_ for Li^+^. These data were interpreted using an ion-pairing model, where ideality is lost at low values of C_crit_ for cations, such as Li^+^, which form strong ion pairs with the pore-wall carbonates [20,26]. This study showed that ion/pore-wall chemistry can play a critical role in determining whether the ideally cation-permselective/cation-conducting state can be achieved.

None of the early studies with gold nanotubes [18,19,23] investigated the effect of the chemical identity of the cation on C_crit_. Thus, it is unknown at this time whether the DLOM always fails (as per the polymeric nanopore membrane) [20] or sometimes prevails. To explore this issue, we prepared gold nanotube membranes [27] where the inside diameter of the tubes was 10 nm, the same as in the polymeric nanopore membrane, hereafter called PC_10_ [20]. Furthermore, like the PC_10_, the gold nanotubes were negatively charged, but the charge was due to attached chloride [18,19,21,28,29,30,31], rather than carbonate.

Potentiometry was used to study the effect of the identity of the cation in the contacting solution on C_crit_ values for this 10 nm diameter gold nanotube membrane. Five monovalent and two divalent cations (chloride salts) were studied. We found that, within experimental error, C_crit_ values for all of these cations agreed with values predicted by DLOM. Thus, these studies show that the ion-pairing chemistry that determines C_crit_ in the PC_10_ membrane is not operative in comparable-sized gold nanotubes, and, as a result, there is no chemical effect on C_crit_. This allows the purely electrostatic DLOM to prevail.

## 2. Materials and Methods

### 2.1. Materials

Commercially available polycarbonate (PC) filtration membranes, prepared by the well-known track-etch method [32], were obtained from Whatman (Marlborough, MA, USA). These membranes are available with pore-diameters as small as 10 ± 2 nm. Membranes with 10 nm pores, called PC_10_, were investigated in a previous study [20]. The membranes used for the experiments described here had 30 ± 1 nm diameter pores and are called PC_30_. These membranes were 6 μm thick and contained 6 × 10^8^ pores cm^−2^. The gold nanotubes were deposited within the pores of this membrane (see below). KCl, KBr, NaCl, MgCl_2_, CaCl_2_, NH_4_Cl, HNO_3_, H_2_SO_4_, and methanol were of reagent grade and purchased from Fisher Scientific (Waltham, MA, USA). Commercial gold plating solution (Oromerse SO Part B) was obtained from Technic, Inc (Cranston, RI, USA). HPLC-grade purified water was obtained from Fisher Scientific and was used for rinsing and to prepare all solutions. All other chemicals were of reagent grade and were used as received from Sigma-Aldrich (St. Louis, MO, USA). All experiments conducted in this work were at a room temperature of 25 °C.

### 2.2. Electroless Plating and Measurement of Nanotube Inside Diameter

**Figure 1:**
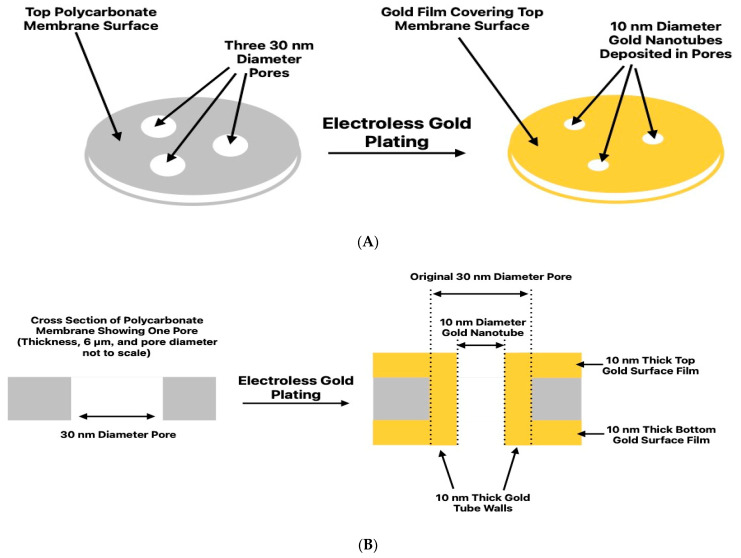
Schematic illustrations of the gold electroless plating process used to make the PC_30,10_ membrane. (**A**) Top and (**B**) cross-sectional views. Dimensions are not to scale.

An electroless plating method, described in detail previously, was used to deposit the gold nanotubes along the pore walls of the PC_30_ [21,27]. The inside diameter of the tubes obtained scale inversely with deposition time. In addition to the gold nanotubes lining the pore walls, this method yielded thin gold surface films on both faces of the membrane [21,27] (Figure 1). Transmission electron microscopy has been used to characterize such tubes [18]. A good review of this electroless gold deposition method can be found in [21], which also discusses methods to measure the inside diameters and diameter distributions of nanotubes.

An electrochemical method was used to measure the inside diameters of the Au nanotubes [33]. Briefly, the nanotube membrane was mounted in a U-tube cell with 0.1 M KCl and a Ag/AgCl wire placed in each half-cell. Using a Keithley 6487 (Cleveland, OH, USA), a voltage sweep was applied across the membrane, and the resulting current was measured. The applied voltage was scanned from −0.2 to 0.2 V, with a step time of 300 s. A long step time was used because the gold nanotubes make the membrane capacitance high, resulting in long decay times for the charging current after each step (see Figure S2). The slopes of the current–voltage (I–V) curves were used to calculate the tube’s inside diameter [33], which was determined to be 10 ± 1 nm. This membrane, with 30 nm diameter pores and containing 10 nm inside-diameter gold nanotubes, is called the PC_30,10_ here. The electrochemical method was also used to measure the pore diameters in the PC_30_. A value of 30 ± 1 nm was obtained after correction for the resistances of the contacting solutions. See Figure S1 and Equation (S1) for details.

### 2.3. Potentiometric Measurements and Methods

As has been discussed in detail previously, exposure of gold nanotube membranes to aqueous chloride salts causes the adsorption of Cl^−^ to the gold tube walls and membrane faces [18,19,21]. Furthermore, adsorption of Cl^−^ to gold electrodes has been extensively investigated in the electrochemical literature [28,29,30,31]. These studies have shown that gold and chloride form a covalent, chemisorption bond [28], and, once adsorbed, negative voltages must be applied to the electrode to strip the Cl^−^ off.

The majority of the potentiometric data were obtained using chloride salts of various monovalent and divalent cations. Prior to potentiometric measurements, the PC_30,10_ was immersed into a 2 M aqueous solution of the desired salt for 8 h, rinsed with water, and then immersed for 16 h in a 0.1 mM solution of that salt. Exposure to Cl^−^ in this way caused Cl^−^ to adsorb to the gold [28,29,30,31], and these surface-bound chlorides provided the negative charge needed to render the PC_30,10_ cation permselective [18,19,21,28,34,35]. To study the effect of anion, potentiometric data were also obtained for KBr solutions. In this case, the PC_30,10_ was exposed to 2 M KBr, which resulted in the adsorption of Br^−^ to the gold [31].

Potentiometric data were obtained using a U-tube cell, where the PC_30,10_ separated two salt solutions, both initially 0.1 mM, designated here, C_l_ [20]. Standard additions of the same salt were added to one solution while maintaining the other at 0.1 mM. A range of high-side concentrations of C_h_, from 0.1 mM to 10 mM, were used. For the Cl^−^ salts, Ag/AgCl electrodes were used to measure the PC_30,10_ voltage, E_mem_ [20]. When a Br^−^ salt was used, Ag/AgBr electrodes were used. An Accumet XL 15 pH meter (Waltham, MA, USA) with a resolution of 0.1 mV was used.

If a membrane is ideally cation permselective, E_mem_ is given by the concentration-cell form of the Nernst equation,

(1) $$E_{mem}=-\frac{0.0592}{z_{+}}\log\frac{a_{{+}_{h}}}{a_{{+}_{l}}}$$

where ${a_{+}}_{h}$ and $a_{{+}_{l}}$ are the activities of the cation in the high and low salt-concentration solutions, respectively, on either side of the membrane, z_+_ is the charge of the cation, and 0.0592 is a collection of constants, including the Kelvin temperature [18,36,37]. Equation (1) predicts that a plot of E_mem_ vs. the log of the activity ratio (a Nernst plot) [20] will be linear with a slope of −59.2 mV per decade change in activity ratio for monovalent cations and half this for divalent cations. We obtained positive slopes by simply reversing the leads of the voltmeter; i.e., the red lead was attached to the reference electrode in the C_l_ solution, and the black to electrode in the C_h_ solution.

If the Nernst plot shows the ideal, Nernstian slope (Equation (1)), the membrane transports only cations and rejects anions and salt [18,19,20]. A sub-Nernstian slope indicates that cations, anions, and salt are now being transported. In this case, the cation transference numbers, t_+_, the fraction of charge carried through the membrane by cations, can be calculated as follows [38,39]:

(2) $$\frac{slope}{0.0592\times z_{+}}=2t_{+}-1$$

where “slope” is the experimentally measured sub-Nernstian slope.

As discussed above, C_crit_ is the C_h_ value above which the Nernst plot becomes non-linear, with the experimental points falling below the extrapolated straight line. C_crit_ was determined by least squares analysis of the Nernst plot data [20]. This analysis yielded the highest concentration that still gives a linear response. C_crit_ was obtained by averaging this last linear concentration and the first concentration that had fallen off the line [20]. Again, it is important to emphasize that at C_h_ above C_crit_, ideal cation permselectivity has been lost, and that predicting the value of C_crit_ is the point of the DLOM.

### 2.4. Surface Contact Angle

Water contact angle measurements were used to study the adsorption of chloride on the PC_30,10_ surface. A Tantec CAM-Plus contact angle meter (Schaumburg, IL, USA) employing the half-angle technique was used. The full contact angles are reported here. Each reported contact angle is the average of ten measurements, five for each membrane face. The PC_30,10_ was exposed to a 2 M aqueous solution of NaCl for 24 h, rinsed, then immersed in DI water for 24 h, and finally, dried in air overnight.

### 2.5. X-ray Photoelectron Spectroscopy (XPS)

XPS was used to study the surface composition of the PC_30,10_ before and after exposure to NaCl. A ULVAC-PHI 5000 Versaprobe-II XPS system (Chigasaki, Japan) equipped with an Al monochromatic source (50 W, 200 µA, takeoff angle 45°) was used. Data analysis was performed using the PHI Multipak software (Version 9.9.0). To calibrate the binding energies, the major carbon 1s peak was set to 284.8 eV [40]. Full survey spectra were obtained with an analyzer pass energy of 187.5 eV and a step size of 0.8 eV. XPS data were obtained on the PC_30,10_ before and after exposure to aqueous 2 M NaCl. Exposure entailed the immersion of the PC_30,10_ into water for 24 h, immersion in 2 M NaCl for 24 h, immersion again in water for 24 h, and then air drying.

## 3. Results and Discussion

### 3.1. Dimensions of the Nanotubes and Surface Films

As shown schematically in Figure 1, electroless deposition yields gold nanotubes lining the pore walls and gold surface films on both membrane faces. The nanotube’s inside diameter was measured both with the Au surface films intact (Figure S1) and after the removal [41] of these films (Figure S1B). A value of 10 ± 1 nm was obtained in both cases (Figure S1B). This is important because it shows that surface bottle-necking of the deposited gold [42] does not occur. This means that the rates of gold plating on the membrane surface and along the pore walls are the same, suggesting no significant differences in the tube-wall and surface-layer gold deposits. Since the inside tube diameter was 10 nm and the outside diameter was 30 nm (PC_30_ pore diameter), the tube wall thickness was 10 nm. Because the surface and tube-wall plating rates are the same, the thickness of the Au surface films was also 10 nm. Finally, a microscopic investigation of electroless gold films showed that the very thin films deposited were fairly compact and morphologically uniform [43].

### 3.2. Surface Contact Angle and XPS Measurements

The unmodified PC_30_ membrane has a water contact angle of 80 ± 4° (Figure S3). After the deposition of the gold nanotubes, the contact angle increased to 130 ± 4°, indicating that the PC_30,10_ surface is quite hydrophobic. By comparison, the contact angle of a clean gold surface is 65 ± 3° [44,45]. Part of this enhanced hydrophobicity of the PC_30,10_ surface was due to the roughness of the gold surface film. Indeed, measured contact angles for intentionally roughed surfaces were as much as 15 degrees higher than for the comparable flat surface [46,47]. We are currently further investigating this unusually large contact angle. However, after exposure to 2 M NaCl, the contact angle decreased to 70 ± 3°. This value is comparable to that of clean gold [44,45] and shows, as would be expected, that adsorption of Cl^−^ renders the surface PC_30,10_ hydrophilic. All measurements were made on this hydrophilic, low contact angle, version of the PC_30,10_.

**Figure 2:**
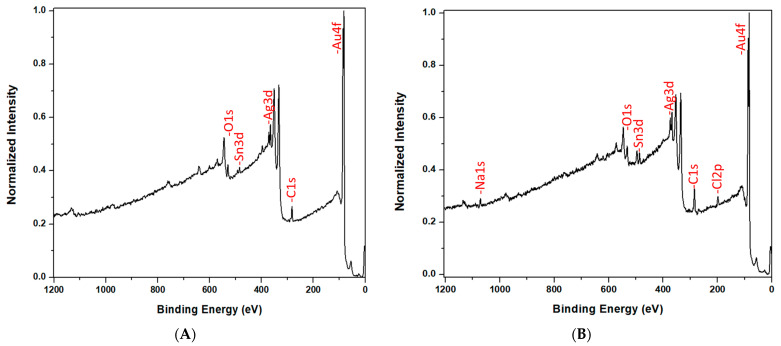
XPS survey spectra of the PC_30,10_ before (**A**) and after (**B**) exposure to 2 M NaCl.

XPS was used as an additional method to confirm that Cl^−^ adsorption occurs to the PC_30,10_ (see Figure 2 below). Before exposure to 2 M NaCl, the most abundant signals in the XPS survey spectrum were from Au, Ag, Sn, C, and O. The surface atom percentages are shown in Table 1. The carbon signal comes from contamination and is typically observed, at high surface percentages, in XPS spectra of metals that have been exposed to air [48,49]. Because of this contamination, only about 30% of the surface was bare gold (Table 1). The Sn and Ag are byproducts of the electroless plating process [27] and are only seen in ultrathin films, such as the 10 nm films plated here. High resolution XPS data show that Ag was present as a metal and Sn was present as SnO_2_, not as a metal (see Supplementary Materials).

After exposure to 2 M NaCl, the PC_30,10_ showed the previous five atoms, plus two new signals, due to Cl and Na each constituting 5 ± 2% of the surface. The Cl signal was the adsorbed Cl^−^ responsible for the decrease in contact angle after exposure to NaCl. The Na signal was from the Na^+^ that must be present on the surface to balance the adsorbed Cl^−^ so as maintain surface electroneutrality. As would be expected, the surface percentages of the Na and Cl signals were the same. Steinle et al. obtained similar results in studies on the adsorption of hydrophobic anions to hydrophobic gold nanotubes, where each adsorbed anion brought a charge-balancing cation to the surface [50].

### 3.3. Nernst Plots and C_crit_

**Figure 3:**
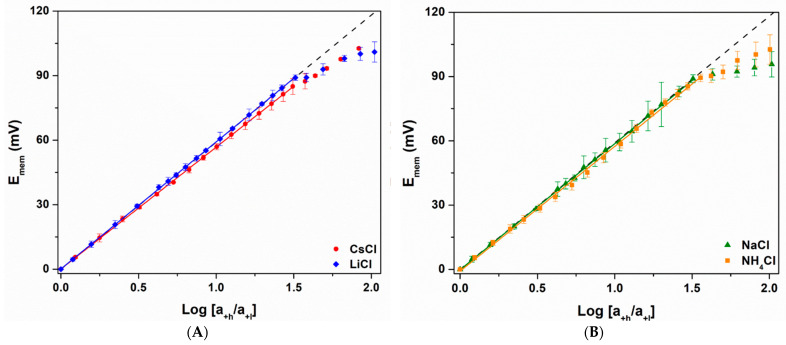
Nernst plots for the PC_30,10_ in a concentration cell containing (**A**) CsCl (red) and LiCl (blue), and (**B**) NaCl (green) and NH_4_Cl (orange). The dashed lines correspond to ideal cation permselectivity (Equation (1)). Standard deviations were determined from three replicate measurements.

**Figure 4:**
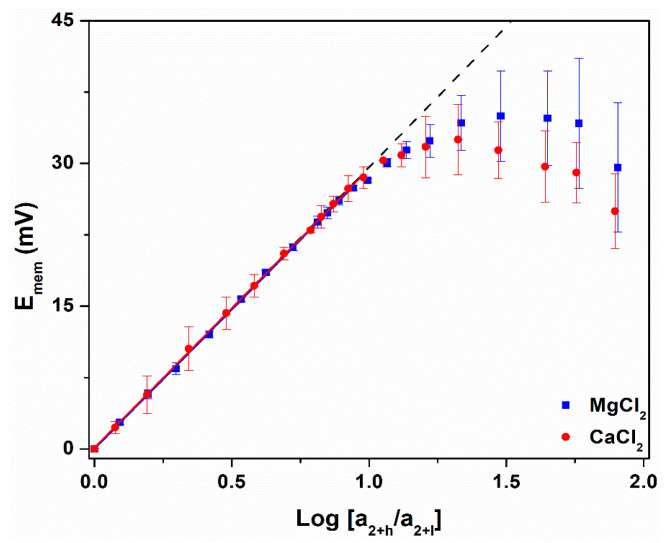
Nernst plots for the PC_30,10_ in a concentration cell containing MgCl_2_ (blue) and CaCl_2_ (red). The dashed line corresponds to ideal cation permselectivity (Equation (1)). Standard deviations were determined from three replicate measurements.

Nernst plots (Figure 3 and Figure 4) show how the membrane voltage, E_mem_, changes with salt concentration differences across the membrane. Because the low-concentration side was held constant at C_l_ = 0.1 mM, it is convenient to express the concentration difference by simply stating the high-side concentration, Ch. The majority of the data were obtained using chloride salts of the various cations and Ag/AgCl wire electrodes [20]. However, to investigate the effect of anion, Nernst plots were also obtained using KBr solutions and Ag/AgBr electrodes. The KCl and KBr Nernst plots were identical (Figure S5).

All Nernst plots showed a region of linear behavior at lower values of C_h_ (C_h_ < C_crit_), followed by a non-linear region at C_h_ > C_crit_ (Figure 3 and Figure 4, Table 2 and Table 3). Similar Nernst plots were obtained in prior investigations of gold nanotube membranes, however KCl was the only electrolyte studied [18,19]. The PC_10_ cation permselective nanopore membrane, where the negative surface charge density is due to carbonate, also showed similar plots [20]. However, in contrast to the PC_10_ [20], we have found that C_crit_ for all five monovalent cations were, within experimental error, identical for the PC_30,10_ (Table 2), with an average value of 3.6 ± 0.2 mM. The divalents showed lower, but again, identical C_crit_ values.

Furthermore, according to DLOM [18,24], C_crit_ is the value of C_h_ where the Debye length, λ_d_ [24,25], for the C_h_ solution is equal to the tube radius (ideal values in Table 2 and Table 3). If this is correct, in the presence of a strong electrolyte with a monovalent cation and anion, the PC_30,10_ (tube radius = 5.0 ± 0.5 nm) should show C_crit_ = 3.7 mM where λ_d_ = 5.0 nm (Figure 3) [18,20,25]. This ideal value is, within experimental error, identical to the average C_crit_ for the monovalents. The experimental C_crit_ for the divalents is also essentially identical to the ideal value (Table 3). If there is error here, it is most likely in the calculated ideal value of C_crit_. This is because the calculation uses the Poisson–Boltzmann equation, which has been shown to be problematic for anything but monovalent salts [26,51,52,53].

It is also interesting to note that Nernst plots for the divalent cations show maximum E_mem_ values, and that E_mem_ decreases with increasing activity ratio above these maxima (Figure 4). This was not observed in the Nernst plots for the monovalents (Figure 3). As shown in our prior work [20], a Nernst plot will show a maximum value of E_mem_ when C_crit_ is low, e.g., 0.9 mM for the divalents (Table 2) vs. 3.6 mM for the monovalents (Table 3). This is because bulk salt transport commences above C_crit_, and when C_crit_ is low, salt incursion causes the value of C_l_ to increase above the initial, and assumed constant, 0.1 mM value [20,54]. This makes the real, i.e., experimental, concentration difference across the membrane less than the assumed value, and, as a result, spuriously low E_mem_ values are obtained. This bulk mixing process at C_h_ > C_crit_ also causes the error bars in the E_mem_ values to become larger than in the ideally permselective range at C_h_ < C_crit_ (Figure 4). Given the size of the error bars in the E_mem_ values, the maxima are experimentally indistinguishable.

That the experimental C_crit_ values for the PC_30,10_ agree with the ideal values indicates that for the five monovalent cations and two divalents studied here, the surface–chloride/cation interaction is purely electrostatic. Again, this can be contrasted with the PC_10_, where non-electrostatic, ion-pairing interactions with the surface carbonates occur. That the surface chlorides in the PC_30,10_ do not engage in ion-pairing is not surprising, given that Cl^−^ in aqueous solution is a poor ion-pairing agent for the cations studied here [26,55,56]. Furthermore, simulations show that an adsorbed Cl^−^ introduces an image charge in the gold and that both the negative charge of the Cl^−^ and the positive image charge are distributed over multiple Au atoms [30,57]. This effectively delocalizes the negative charge relative to a Cl^−^ in solution. This makes a PC_30,10_ surface chloride an even weaker ion-pairing agent than a solution Cl^−^. This is why the surface chlorides in the PC_30,10_ do not engage in ion pairing and why the purely electrostatic, double-layer overlap model prevails.

Finally, some cations, most notable Cs^+^, showed very slightly sub-Nernstian slopes. The extent of this non-ideality can be quantified by using Equation (2) to calculate the cation transference numbers, t_+_, from the ratio of the experimental-to-ideal slopes. The t_+_ data show that even the most sub-Nernstian ion, Cs^+^ (t_+_ = 0.978), gives nearly ideal responses in the linear region (Table 2). We have recently investigated the apparent small non-ideality observed for Cs^+^ in much smaller inside-diameter gold nanotubes and will have more to say about this in a future publication.

## 4. Conclusions

This work is part a broader research effort to explore how ion/surface chemistry affects ion-transport through an ionic composition of nanoscale-confined solutions [18,20,21]. Results with the PC_10_ membrane showed that ion-pairing between cations and the pore wall carbonates cause ideality to be lost at low salt concentrations. From a practical point of view, this is undesirable since, again, the ideal state produces optimal device performance in many proposed nanofluidic devices [4,6,58,59]. It is worth mentioning that when organic cations are used, hydrophobic interactions cause the adsorption of these cations to the PC_10_ pore walls [54]. As a result, ideality could not be achieved under any experimental conditions for any of the organic cations studied [54].

The overriding conclusion is that in order to achieve maximum efficiency, ion/surface chemical interactions must be eliminated or minimized. As demonstrated here, this can be accomplished by using a fixed negative group, such as adsorbed chloride, that is a very weak Lewis base. Sulfonate would be another good choice. Unfortunately, the carbonate in the PC_10_ was not a weak Lewis base, nor is carboxylate, which is often attached to carbon nanotubes [13,20]. Finally, if organic cations or anions are involved in the process, then pores/tubes/channels that are based on hydrophilic materials would be preferable [60].

## Figures and Tables

**Table 1:** XPS surface composition for the PC_30,10_ before and after exposure to 2 M NaCl. The atoms shown were the most abundant.

Element Peak	PC_30,10_, Control (%)	PC_30,10_, NaCl ^1^ (%)
C1s	32.4	48 ± 3
Au4f	46.9	25 ± 3
O1s	16.0	12 ± 3
Ag3d	4.0	3.1 ± 0.5
Sn3d	0.8	0.9 ± 0.2
Cl2p	-	5 ± 2
Na1s	-	5 ± 2

^1^ Averages and standard deviations are from three identical samples.

**Table 2:** Experimental data from the Nernst plots for the monovalent ions (Figure 3).

Cation	Slope of Linear Response Region (mV)	t_+_ of Linear Response Region	C_crit_ (mM)
Ideal	59.2	1.000 *^a^*	3.7 *^b^*
Li^+^	59.1 ± 0.1	0.999 ± 0.001	3.5 ± 0.1
Na^+^	58.8 ± 0.3	0.997 ± 0.003	3.7 ± 0.1
K^+^	58.5 ± 0.2	0.994 ± 0.002	3.5 ± 0.2
NH_4_^+^	58.2 ± 0.6	0.992 ± 0.005	3.8 ± 0.1
Cs^+^	56.6 ± 0.2	0.978 ± 0.002	3.4 ± 0.3

*^a^* Ideal t_+_ was calculated from Equation (1) for an ideal slope of 59.2 mV. Experimental t_+_ values were calculated from Equation (2) and the experimental slope. *^b^* Concentration of salt where the calculated [25] Debye length is equal to the tube radius, 5.0 nm. Standard deviations were determined from three replicate measurements.

**Table 3:** Experimental data from the Nernst plots for the divalent ions (Figure 4).

Cation	Slope of Linear Response Region (mV)	t_+_ of Linear Response Region	C_crit_ (mM)
Ideal	29.6	1.000 *^a^*	1.2 *^b^*
Ca^2+^	29.4 ± 0.2	0.997 ± 0.003	0.9 ± 0.2
Mg^2+^	29.3 ± 0.1	0.995 ± 0.002	0.9 ± 0.1

*^a^* Ideal t_+_ was calculated from Equation (1) for an ideal slope of 29.6 mV. Experimental t_+_ values were calculated from Equation (2) and the experimental slope. *^b^* Concentration of salt where the calculated [25] Debye length is equal to the tube radius, 5.0 nm. Standard deviations were determined from three replicate measurements.

## Data Availability

The original contributions presented in this study are included in the article/Supplementary Materials; further inquiries can be directed to the corresponding author/s.

## Supplementary Materials

The following supporting information can be downloaded at https://www.mdpi.com/article/10.3390/nano14141209/s1. S1: Measurement of Gold Pore/Tube Diameter. Figure S1: Current–voltage curves of 0.1 M KCl through PC_30_ and PC_30,10_ with and without gold surface layers. S2 and Figure S2: Decay Times of Charging Currents during Pore Diameter Measurements. S3 and Figure S3: Photographic Image of Water Contact Angle on PC_30_. S4: XPS analysis of Silver and Tin Gold-Plating Byproducts. Figure S4: High Resolution XPS Spectra of Silver and Tin Gold-Plating Byproducts. S5 and Figure S5: Nernst plots for KCl and KBr. References [61,62,63] are used to support analysis of the gold-plating byproducts.
