# Supplementary material for: Potentiometric Studies on Ion-Transport Selectivity in Charged Gold Nanotubes
Source: Nanomaterials (Basel). 2024 Jul 16;14(14):1209. doi: 10.3390/nano14141209 (PMC11280230; doi:10.3390/nano14141209)
Supplement: Supplementary file 1 [file nanomaterials-14-01209-s001.zip › nanomaterials-3056336-supplementary.pdf]

Supporting Information

Potentiometric Studies of Ion-Transport Selectivity in Charged Gold Nanotubes

Thomas T. Volta [1], Stevie N. Walters [1], and Charles R. Martin [1,*]

[1] Department of Chemistry, University of Florida, Gainesville, FL 32611-7200, USA

[*] crmartin@chem.ufl.edu

Table of Contents

S1. Measurement of Gold Pore/Tube Diameter 2
S2. Decay Times of Charging Currents During Pore Diameter Measurement 3
S3. Water Contact Angle of PC_{30} 4
S4. XPS Analysis of Silver and Tin Gold-Plating Byproducts 5
S5. Nernst Plots for KCl and KBr 6
S6. References 6

S1. Measurement of Gold Pore/Tube Diameter

The diameters of the nanotubes in gold-plated $PC_{30,10}$ were calculated from current-voltage (I-V) curves by,

$$\boldsymbol{d} = \mathbf{2}\sqrt{\frac{\boldsymbol{lG}}{\boldsymbol{\pi A N \kappa}}} \qquad \text{(S1)}$$

where d is the nanopore diameter in cm; l is the length of the pore (0.0006 cm); G is the conductance in Siemens (slope of the curve); A is the membrane area in cm^2; N is the pore density (6×10^8 pores cm^{-2}); and κ is the conductivity of 0.1 M KCl in units of Siemens per cm [1,2]. Figure S1A shows the resulting I-V curves for the membrane before (black) and after (red) gold-plating.

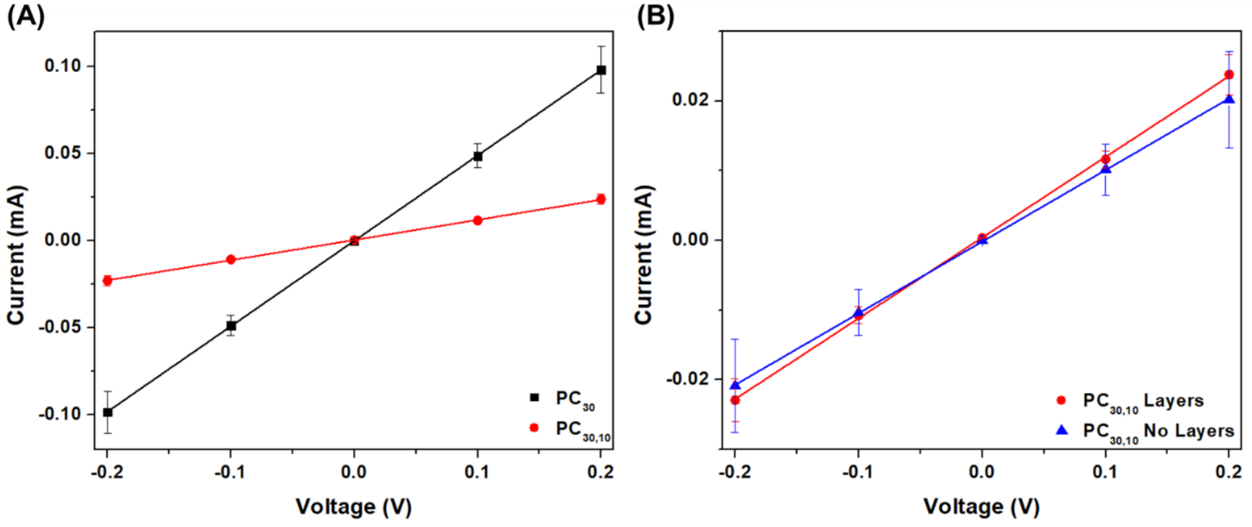

Figure S1. Current-voltage curves of 0.1 M KCl through (A) PC_{30} (black) and $PC_{30,10}$ (red) with gold surface layers. Displayed current-voltage curves are before correction for the contribution of the resistances of the contacting solutions. (B) Current-voltage curves of $PC_{30,10}$ with (red) and without (blue) gold surface layers. Standard deviations were determined from ten replicate measurements of three separate membranes each.

After correcting for the resistances of the contacting solutions, PC_{30} was found to have a nanopore diameter of 30 ± 1 nm, while gold-plated $PC_{30,10}$ was found to have a nanotube diameter of 10 ± 1 nm. PC_{30} and $PC_{30,10}$ rectification ratios of 1.00 ± 0.01 and 0.97 ± 0.01, respectfully, were acquired indicating that the nanotubes retained their original, cylindrical shape after gold-plating [1]. The rectification ratio is defined by the current measured at -0.2 V divided by the current at 0.2 V [1].

To prove that the rate of gold plating is the same on the membrane faces as it is in the nanopore, I-V curves of $PC_{30,10}$ with and without the gold surface films were obtained (Figure S1B). If the plating rate is faster at the membrane surface, a smaller tube diameter would be obtained from the current-voltage curve of $PC_{30,10}$ with the surface films intact. However, from Figure S1B and Equation S1, the same nanotube diameter is obtained from $PC_{30,10}$ with (red) and without (blue) the gold surface films. This verifies that no bottle-necking occurred [3].

S2. Decay Times of Charging Currents During Pore Diameter Measurement

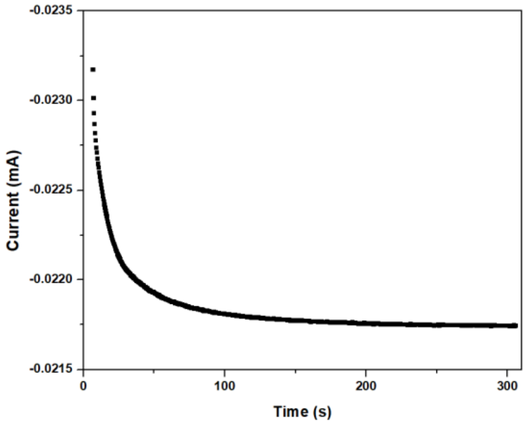

(**A**)

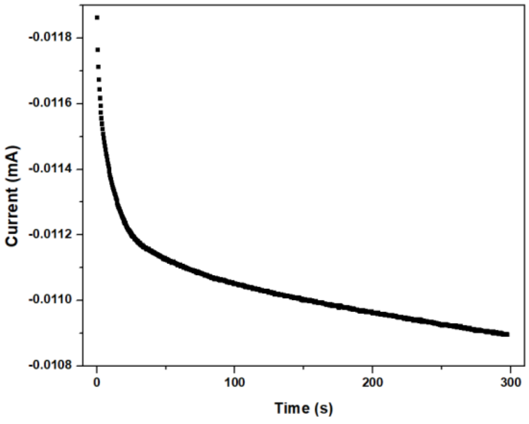

(**B**)

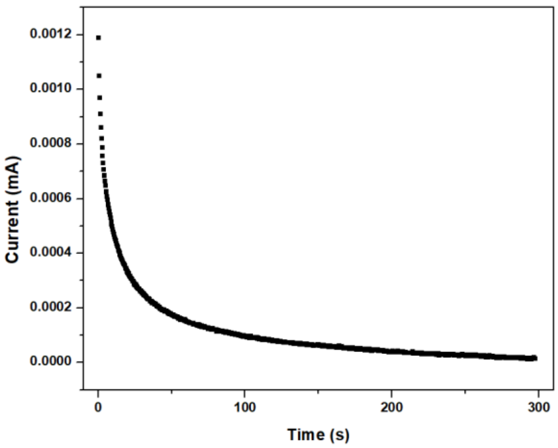

(**C**)

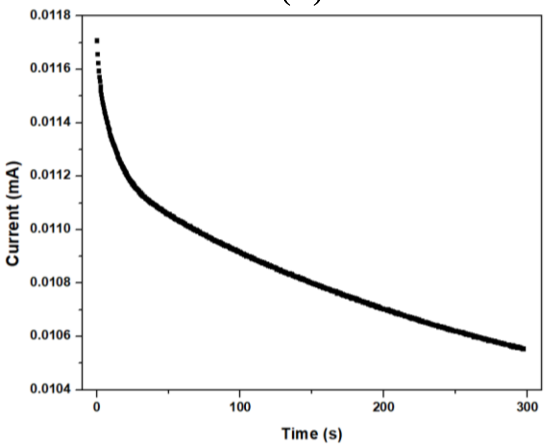

(**D**)

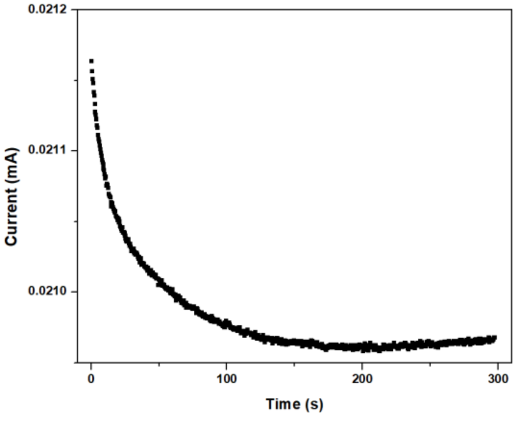

(**E**)

Figure S2. Decay times of charging current during pore diameter measurement at (A) -0.2 V (B)-0.1 V (C) 0.0 V (D) 0.1 V and (E) 0.2 V.

For some of the transients the charging currents are still decaying away at 300 seconds. However, in all cases, greater than 90% of the charging current had decayed away, as calculated from the zero-time (maximum) current and the 300 seconds (measured) current. To show that further discharge does not affect the measured tube diameter, we calculated the tube diameter from the current at a shorter time, 200 seconds, where less of the charging current had decayed away. The value we get from the 200 second calculation, 9.6 nm, is within the experimental error of the value calculated from the 300 second data, 10 $\pm$ 1 nm. Given this 10% experimental error, allowing the currents to decay further will not change the measured tube diameter.

S3. Water Contact Angle of PC_{30}

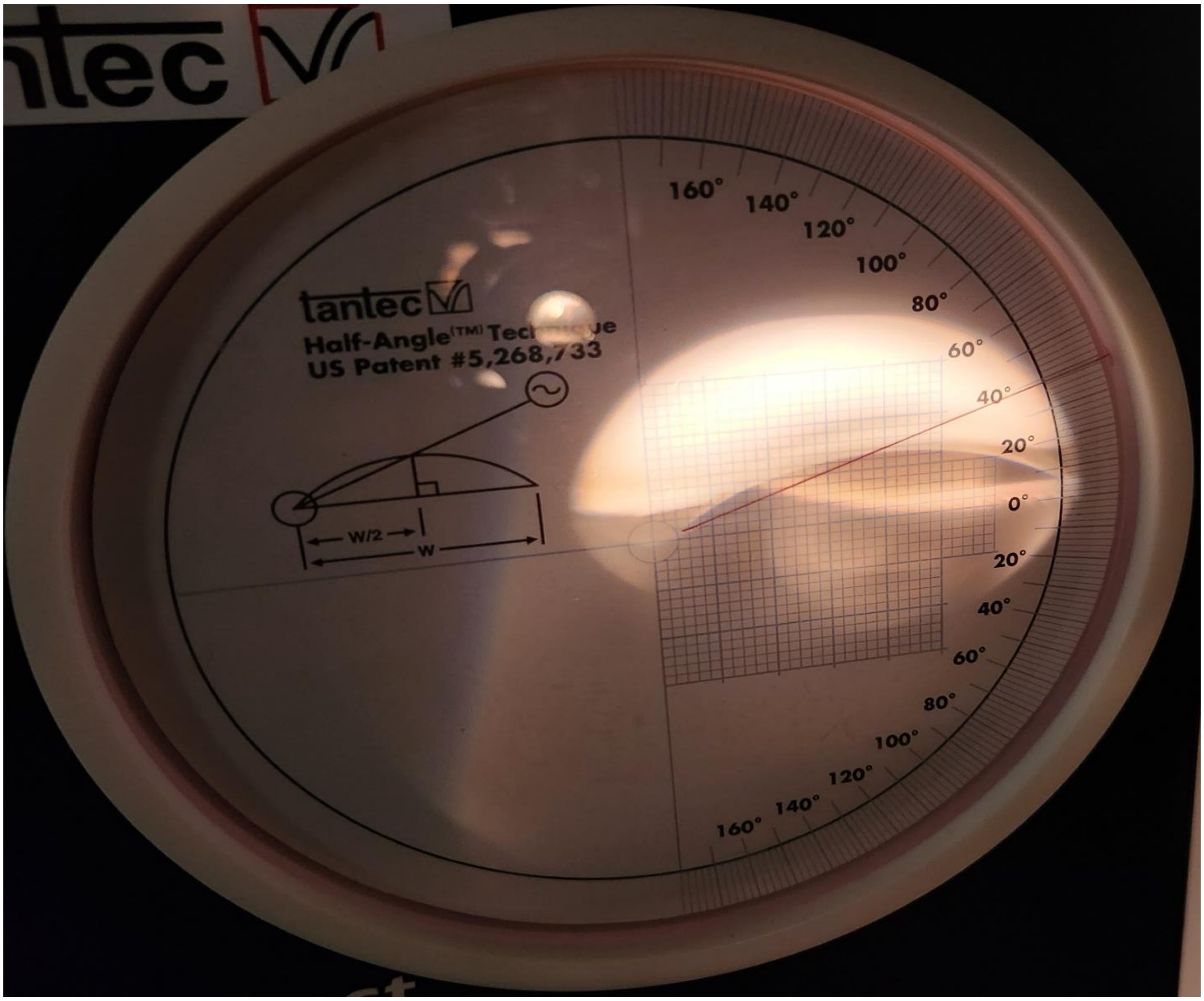

Figure S3. Photographic image of water contact angle on a PC_{30} membrane, using the half-angle technique. A total of 10 replicate measurements were made, 5 for each membrane face. The full (not half) contact angle was determined to be 80 $\pm$ 4°.

S4. XPS Analysis of Silver and Tin Gold-Plating Byproducts

XPS was used to study the nature of the Ag and Sn contaminates leftover from the gold-plating process. A ULVAC-PHI 5000 Versaprobe-II XPS system equipped with an Al monochromatic source (50 W, 200 µA, takeoff angle 45°) was used. To calibrate binding energies, the carbon 1s peak was set to 284.8 eV [4]. High resolution spectra were obtained with an analyzer pass energy of 23.5 eV and a step size of 0.1 eV.

The Ag spectrum shows two peaks centered at the binding energies of 368.3 and 374.4 eV with a chi squared best fit of 1.33. These correspond to the $3d_{5/2}$ and $3d_{3/2}$ orbitals. When Ag forms an oxide (either Ag_2O or AgO) shifts of approximately -0.3 and -0.8 eV are observed [5]. The silver metal $3d_{5/2}$ orbital binding energy is centered around 368.2 eV [6]. The $3d_{5/2}$ orbital of silver did not shift to lower binding energies when measured on the $PC_{30,10}$ membrane. This confirms that the silver contaminate is present as metal Ag nanoparticle and not an oxide.

The Sn spectrum shows two peaks centered at the binding energies of 487.6 and 496.0 eV with a chi-squared Gaussian fit of 1.05. These also correspond to the $3d_{5/2}$ and $3d_{3/2}$ orbitals. The Sn metal $3d_{5/2}$ orbital binding energy is found to be centered around 485.0 eV [6]. Large ~2.6 eV shifts are seen between the 3d doublet of Sn metal and Sn oxides. These binding energies are in agreement with the literature reported values for SnO_2 [7]. This confirms the Sn contaminate is not tin metal but rather tin dioxide.

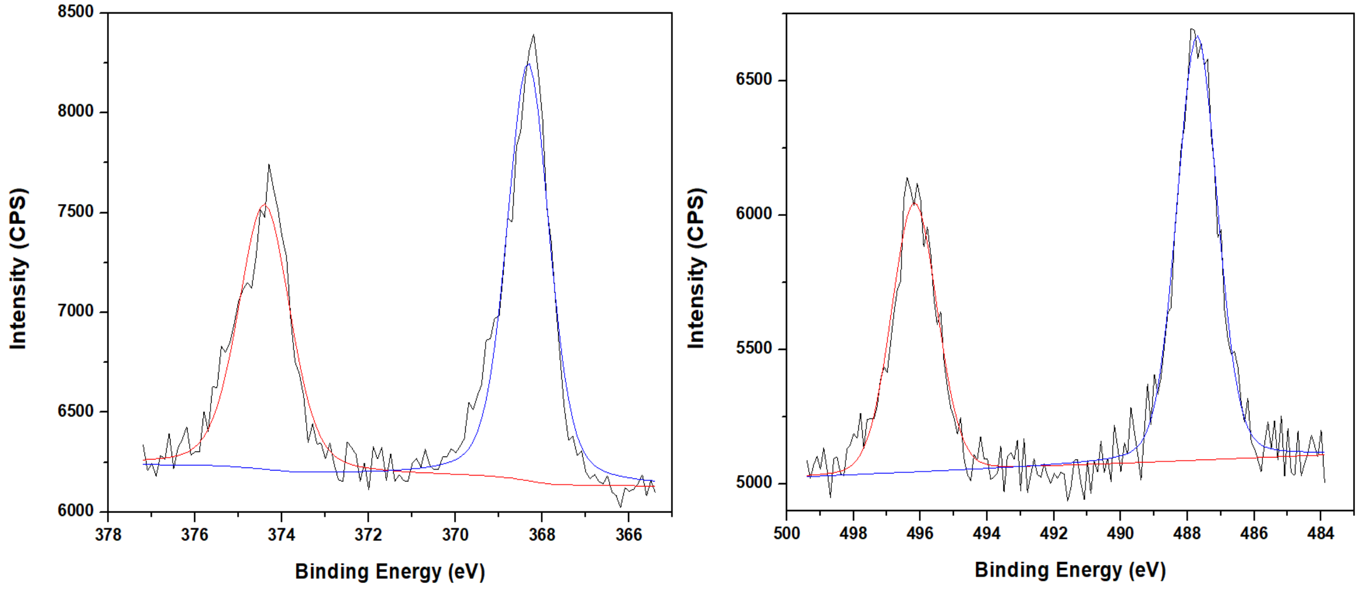

(A) (B)

Figure S4. High resolution XPS spectra of (**A**) silver and (**B**) Tin. The solid black lines are the experimental data. Red and blue solid lines are best Gaussian fits of the experimental data.

S5. Nernst Plots for KCl and KBr

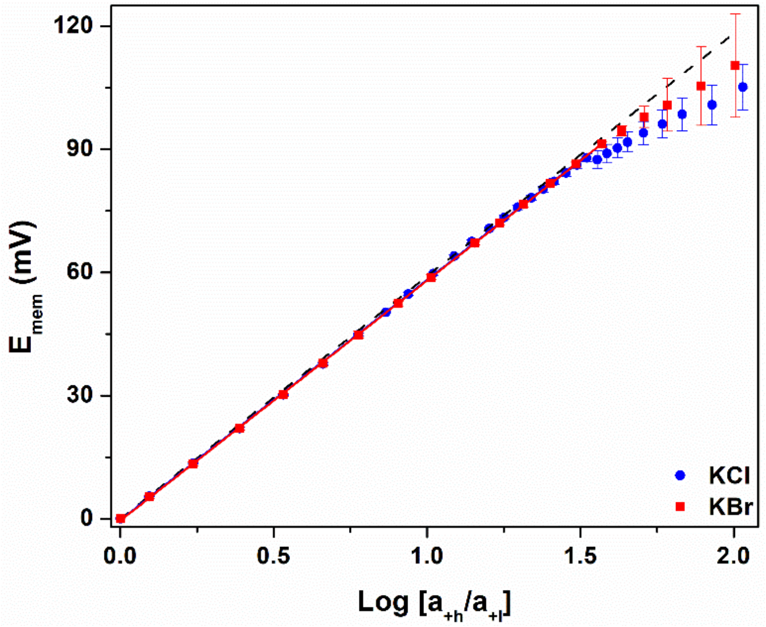

Figure S5. Nernst plots for $PC_{30,10}$ in a concentration cell containing KCl (blue) and KBr (red). The dashed line corresponds to ideal cation permselectivity (Equation 1). Standard deviations were determined from three replicate measurements.

S6. References

1. Bush, S.N.; Ken, J.S.; Martin, C.R. The Ionic Composition and Chemistry of Nanopore-Confined Solutions. *ACS Nano* **2022**, *16*, 8338-8346, doi:10.1021/acsnano.2c02597.
2. Harrell, C.C.; Lee, S.B.; Martin, C.R. Synthetic Single-Nanopore and Nanotube Membranes. *Analytical Chemistry* **2003**, *75*, 6861-6867, doi:10.1021/ac034602n.
3. Jirage, K.B.; Hulteen, J.C.; Martin, C.R. Nanotubule-Based Molecular-Filtration Membranes. *Science* **1997**, *278*, 655-658, doi:10.1126/science.278.5338.655.
4. Jacquemin, M.; Genet, M.J.; Gaigneaux, E.M.; Debecker, D.P. Calibration of the X-Ray Photoelectron Spectroscopy Binding Energy Scale for the Characterization of Heterogeneous Catalysts: Is Everything Really under Control? *ChemPhysChem* **2013**, *14*, 3618-3626. https://doi.org/10.1002/cphc.201300411.
5. Gaarenstroom, S.W.; Winograd, N. Initial and final state effects in the ESCA spectra of cadmium and silver oxides. *The Journal of Chemical Physics* **1977**, *67*, 3500-3506, doi:10.1063/1.435347.
6. Moulder, J.F.; Stickle, W.F.; Sobol, P.E.; Bomben, K.D. *Handbook of X-ray Photoelectron Spectroscopy*; Perkin-Elmer Corporation, Physical Electronics Division: Eden Prairie, MN, 1992.
7. Liu, C.; Félix, R.; Forberich, K.; Du, X.; Heumüller, T.; Matt, G.J.; Gu, E.; Wortmann, J.; Zhao, Y.; Cao, Y.; et al. Utilizing the unique charge extraction properties of antimony tin oxide nanoparticles for efficient and stable organic photovoltaics. *Nano Energy* **2021**, *89*, 106373. https://doi.org/10.1016/j.nanoen.2021.106373.
